# Hydroxychloroquine increases the exposure of methotrexate in plasma and red blood cells: a pharmacokinetic interaction study in rats in vivo

**DOI:** 10.3389/fphar.2025.1561001

**Published:** 2025-04-24

**Authors:** Guijie Zhang, Rui Wang, Geping Chen, Simin Liu, Hongyu Jie, Wenying Chen, Qiang Li

**Affiliations:** ^1^ Department of Pharmacy, The Third Affiliated Hospital of Southern Medical University, Guangzhou, Guangdong, China; ^2^ School of Pharmaceutical Sciences, Southern Medical University, Guangzhou, Guangdong, China; ^3^ Department of Rheumatology and Immunology, The Third Affiliated Hospital of Southern Medical University, Guangzhou, Guangdong, China

**Keywords:** methotrexate, methotrexate polyglutamates, hydroxychloroquine, rheumatoid arthritis, pharmacokinetics

## Abstract

**Introduction:**

Hydroxychloroquine (HCQ) has been demonstrated to be potential to enhance the therapeutic efficacy of methotrexate (MTX) for rheumatoid arthritis (RA) patients. However, the pharmacokinetics (PK) alterations and underlying mechanisms differentiating MTX-HCQ combination therapy from MTX monotherapy remain uncharted.

**Methods:**

Thirty-three Sprague-Dawley rats were divided into single-dose and multiple-dose groups, with each group further randomized into an MTX monotherapy group an Hydroxychloroquine monotherapy group (HTG), and an MTX-HCQ combination therapy group Blood samples were collected at various time points before and after dosing to determine drug concentrations in plasma and red blood cells (RBC). The area under the concentration-time curve (AUC) for each compound was calculated, and pharmacokinetics models were established to analyze parameter variations across groups.

**Results:**

In the single-dose group, the CTG exhibited a significant increase in the RBC MTX Cmax compared to the MTG (P = 0.023), whereas the AUC of RBC MTX showed an increasing trend (P = 0.056). In the multiple-dose group, the CTG demonstrated significant increases in plasma MTX Cmax and AUC (P = 0.023, P = 0.028, respectively) as well as RBC MTX Cmax and AUC (P = 0.010, P = 0.003, respectively). The RBC MTX polyglutamates (MTXPG2 and MTXPG3) also showed an increasing trend in Cmax and AUC for the CTG. Additionally, the CTG displayed a significant reduction in clearance rate (CLe) (P = 0.001). No significant differences were observed in the Cmax or AUC of HCQ or desethylhydroxychloroquine (DHCQ) in plasma or RBC across dosing groups.

**Conclusion:**

These findings provide insights into the enhanced efficacy, faster onset, and prolonged effect of MTX-HCQ combination therapy compared to MTX monotherapy. The observed increases in MTX Cmax and AUC suggest the need for careful monitoring of MTX-related adverse effects, particularly in patients with renal insufficiency, during combination treatment with HCQ.

## 1 Introduction

Rheumatoid arthritis (RA) is a prevalent systemic autoimmune disease characterized by inflammatory responses, chronic synovitis, and bone destruction. Its primary clinical manifestations include progressive joint damage, dysfunction, deformity, and, ultimately, the potential for disability (Combe et al., 2017; Kong and Wen, 2019; Smolen et al., 2016). Methotrexate (MTX) serves as the cornerstone medication for managing RA, achieving treatment objectives related to disease control and symptom alleviation by inhibiting key enzymes within the folate pathway (Friedman and Cronstein, 2019; Visser & Van Der Heijde, 2009). However, in clinical practice, MTX exhibits a slow onset of action, requiring 1–3 months for therapeutic effects to materialize, and there are notable individual variances in its efficacy (Salliot & Van Der Heijde, 2009). Specifically, 30%–40% of patients demonstrate an inadequate response, with some also experiencing adverse reactions (Ebina et al., 2019; Huang et al., 2020; Huerta-García et al., 2021). Although the underlying reasons for these disparities remain to be elucidated, variability in the pharmacokinetics (PK) of MTX and its metabolite, methotrexate polyglutamates (MTXPGs), at the target site concentrations may contribute to the differences in both efficacy and adverse effects.

Hydroxychloroquine (HCQ), a 4-aminoquinoline antimalarial agent, exhibits anti-inflammatory and immunomodulatory properties. Research has shown that HCQ can increase the plasma concentration of MTX, thereby enhancing its bioavailability. The concurrent administration of HCQ and MTX can accelerate the time to onset, improve early-stage efficacy, and maximize the therapeutic benefits of treatment (Carmichael et al., 2002; Schapink et al., 2019). MTX is predominantly eliminated by the kidneys within 24 h, rendering plasma trough concentrations of MTX generally less informative.

Red blood cells (RBC) provide easily accessible specimens, and elevated concentrations of RBC MTXPGs correlate with reduced disease activity (Van De Meeberg et al., 2023). Consequently, RBC MTXPGs serve as reliable biomarkers for assessing treatment efficacy (Takahashi et al., 2017). In the early phases of the study, high-performance liquid chromatography (HPLC) methods were established to quantify the concentrations of various test substances in plasma and RBC, including MTX in plasma, MTXPGs in RBC, and HCQ along with its metabolite desethylhydroxychloroquine (DHCQ) in both plasma and RBC (Gui-jie et al., 2024). Our primary focus is to examine the PK differences among three groups: the MTX monotherapy group (MTG), the HCQ monotherapy group (HTG), and the MTX-HCQ combination therapy group (CTG). This investigation aims to elucidate the interaction between MTX and HCQ in Sprague-Dawley (SD) rats. Additionally, by constructing PK models, we seek to deepen our understanding and analysis of the differences in the processes of drug absorption, distribution, metabolism, and excretion (ADME) in these animal subjects. The ultimate goal is to provide a PK perspective that contributes to the understanding of the accelerated onset and improved efficacy observed with the combination of MTX and HCQ in the clinical management of patients with RA.

## 2 Materials and methods

### 2.1 Drugs and compounds

Methotrexate tablets were sourced from SPH Sine Pharmaceutical Laboratories Co., Ltd., whereas HCQ sulfate tablets were obtained from SPH Zhongxi Pharmaceutical Co., Ltd. Both MTX (purity 99.8%) and HCQ (purity 99.8%) were acquired from the National Institutes for Food and Drug Control of China. The following compounds were purchased from Schircks Laboratories in Switzerland: 4-amino-10-methylpteroyldiglutamic acid (MTXPG_2_, purity 97%), 4-amino-10-methylpteroyltriglutamic acid (MTXPG_3_, purity 97%), 4-amino-10-methylpteroyltetraglutamic acid (MTXPG_4_, purity 97%), and 4-amino-10-methylpteroylpentaglutamic acid (MTXPG_5_, purity 97%). DHCQ was obtained from Panphy Chemical Corporation, and H_2_O_2_ (3%) was procured from Hebei Jianning Pharmaceutical Co., Ltd.

### 2.2 Instruments

The study utilized an Ultimate 3,000 dual ternary HPLC system, equipped with dual ternary gradient pumps, an auxiliary pump, an automatic sampler, a column oven, a diode array detector, and a fluorescence detector. The photochemical reactions were carried out using the PHRE-15 reactor, which features a 254 nm low-pressure mercury ultraviolet lamp and contains 2 m of 1/16-inch (o.d.) Teflon FEP tubing (0.25 mm i.d.), arranged as a knitted coil.

### 2.3 Animals

Male SD rats, weighing between 160 and 200 g, were obtained from the Guangdong Medical Laboratory Animal Center. The animals were housed in an environment maintained at a temperature of 25°C ± 1°C and a relative humidity of 60% for a period of 3–7 days. During this acclimatization period, the rats had *ad libitum* access to water and standard rat food. The project was approved by the Experimental Animal Ethics Committee of Southern Medical University.

### 2.4 Experimental design

The SD rats were divided into three groups: the blank group, the single-dose group, and the multiple-dose group (over 1 month). Methotrexate tablets were dissolved in a solution of 0.9% NaCl: 5% NaHCO_3_ (4:1), whereas HCQ tablets were dissolved in 0.9% NaCl. The dosage and interval of administration of the drug in rats are based on the usage and dosage of the drug in adults, and the conversion is based on the equivalent dose between animals and humans (Reagan-Shaw et al., 2008).

#### 2.4.1 Blank group

Two SD rats were maintained under standard feeding conditions to provide blank blood samples.

#### 2.4.2 Single-dose group

A total of fifteen SD rats were randomly assigned to three groups. Each group received intragastric administrations of: MTX at 3.5 mg/kg (MTG, *n* = 5), HCQ at 40.1 mg/kg (HTG, *n* = 5), or a combination of MTX at 3.5 mg/kg and HCQ at 40.1 mg/kg (CTG, *n* = 5).

#### 2.4.3 Multiple-dose group

Eighteen SD rats were again randomly divided into three groups. The groups underwent weekly intragastric administrations of MTX at 3.5 mg/kg (MTG, *n* = 6), daily administrations of HCQ at 40.1 mg/kg (HTG, *n* = 6), or a combination of MTX at 3.5 mg/kg weekly with HCQ at 40.1 mg/kg daily (CTG, *n* = 6). Additionally, the MTG group received an equivalent volume of saline on days when the drug was not administered.

The rats were administered drugs intragastrically as previously described. An approximate volume of 0.5 mL of blood was collected from the orbital venous plexus of each rat prior to and following drug administration. Blood sampling time points were established at 0, 15, 30 min, 1, 3, 6, 10, 24, 48, 72, and 96 h. The collected blood was transferred into heparinized EP tubes. The samples were subsequently centrifuged at 3.0 × 10^3^ rpm min^−1^ for 5 min to separate plasma and RBC. The RBC were washed twice with three times their volume of cold physiological saline and then stored in a −25°C refrigerator for future analysis.

### 2.5 Bioanalytical methodology

#### 2.5.1 Detection of plasma MTX, RBC MTX and RBC MTXPG_2-5_ concentrations

The concentrations were measured utilizing a methodologically validated high-performance liquid chromatography with fluorescence detection (HPLC-FLD) technique.

The sample pretreatment method is as follows: 1) For plasma samples, 100 μL of plasma was mixed with 10 μL of a water/mixed standard solution of each concentration, thoroughly mixed for 10 s, and subsequently 100 μL of a 10% perchloric acid solution was added to precipitate proteins. The mixture was vortexed for 3 min and then centrifuged at 1.2 × 10^4^ rpm min^−1^ for 10 min at 10°C. A total of 150 μL of the supernatant was transferred to a sample vial for analysis. 2) For RBC samples, 100 μL of freeze-thawed RBC was initially combined with 100 μL of water to reduce viscosity, followed by the addition of 10 μL of a water/mixed standard solution of each concentration. After mixing for 10 s, 200 μL of a 10% perchloric acid solution was added for protein precipitation. This mixture was vortexed for 3 min and centrifuged at 1.2 × 10^4^ rpm min^−1^ for 10 min at 10°C, after which 300 μL of the supernatant was transferred to a sample vial for analysis.

The chromatographic conditions are as follows: The photochemical reactor was connected online following the analytical column and prior to the fluorometric detector. The analytical column utilized was an Athena C_18_-WP (250 mm × 4.6 mm, 3 μm), and the SPE column was a C_18_-WP (20 mm × 4 mm, 5 μm). Mobile phase A_1_ consisted of 10 mM ammonium acetate (pH adjusted to 9.00 with 25% aqueous ammonia), A_2_ contained 10 mM ammonium acetate (pH = 7.00), with both A_1_ and B_1_ being acetonitrile. A 3% hydrogen peroxide (H_2_O_2_) solution was introduced post-column to derivatize the target compounds using an auxiliary pump. A volume of 100 μL was injected for samples processed from plasma, whereas 200 μL was injected for samples processed from RBC. The separation was carried out by a programmed gradient elution scheme. For specific conditions, see Supplementary Table S1-1 in Annex 1.

The lower limits of quantification were determined to be 1.0 nM for MTX in plasma and RBC, and 2.0 nM for MTXPG_2_ and MTXPG_3_ in RBC.

#### 2.5.2 Detection of plasma HCQ, plasma DHCQ, RBC HCQ and RBC DHCQ concentrations

The concentrations were measured using a methodologically validated HPLC approach.

The sample pretreatment method is as follows: 1) For the plasma samples, 100 μL of plasma is taken, followed by the addition of 10 μL of a water/mixed standard solution corresponding to each concentration. The mixture is then thoroughly mixed for 10 s, after which 150 μL of a methanol-copper sulfate solution is added to precipitate proteins. This combined solution is vortexed for 3 min and subsequently centrifuged at 12,000 rpm min^−1^ for 10 min at 10°C. Finally, 150 μL of the supernatant is transferred to a sample vial for analysis. 2) For the RBC samples, 50 μL of freeze-thawed RBC is combined with 10 μL of a water/mixed standard solution of each concentration. The mixture is evenly mixed for 10 s, and then 250 μL of a methanol-copper sulfate solution is added to precipitate proteins. Similar to the plasma samples, the mixed solution is vortexed for 3 min and centrifuged at 12,000 rpm min^−1^ for 10 min at 10°C. A total of 150 μL of the resulting supernatant is transferred to a sample vial for analysis using an ultraviolet detector.

The chromatographic conditions are as follows: The analytical column employed is the Athena C_18_-WP (150 mm × 4.6 mm, 5 μm), whereas the SPE column is the C_18_-WP (20 mm × 4 mm, 5 μm). The mobile phases are defined as follows: A_1_ consists of a 0.006% aqueous phosphoric acid solution (pH = 3.00), A_2_ is a 10 mM ammonium acetate solution (pH = 7.00), and both B_1_ and A_1_ are composed of acetonitrile. For both the plasma and RBC samples processed, 100 μL is injected for analysis. The separation was carried out by a programmed gradient elution scheme. For specific conditions, see Supplementary Table S1-2 in Annex 1.

The lower limits of quantification were determined to be 15.6 ng/mL for both HCQ and DHCQ in both plasma and RBC. The specificity, standard curve and lower limit of quantification, accuracy, precision, and stability of the method were evaluated in accordance with the Guiding Principles for Verification of Quantitative Analysis Methods of 9,012 Biological Samples of China Pharmacopoeia (2020 edition), with all results meeting the established standards.

### 2.6 Statistical analysis

The area under the concentration-time curve (AUC) was calculated using the linear trapezoidal method. The calculation equations are as follows: Equations 1, 2. Among them, ${\text{AUC}}_{0-\text{last}}$ was the AUC from the zero sampling time to the last sampling time, ${\text{AUC}}_{0-\infty }$ is the AUC from the zero sampling time to infinity, $C_{\text{last}}$ was the last measured concentration, and $\lambda_{z}$ was the terminal slope of the log-linear regression.

$${AUC}_{0-last}=\int_{0}^{last}C\left(t\right)dt$$ (1)

$${AUC}_{0-\infty }={AUC}_{0-last}+\frac{C_{last}}{\lambda_{z}}$$ (2)

The selection of statistical tests (independent samples t-test, Welch’s t-test, or Mann-Whitney U test) was based on the normality distribution of the data and the results of homogeneity of variance testing. Appropriate analytical methods was applied to assess potential differences in the PK parameters of MTX between the MTG and CTG groups. Similarly, this methods was employed to investigate differences in the PK parameters of HCQ between the HTG and CTG groups, with statistical significance determined at p ≤ 0.05.

### 2.7 PK model establishment

**FIGURE 1:**
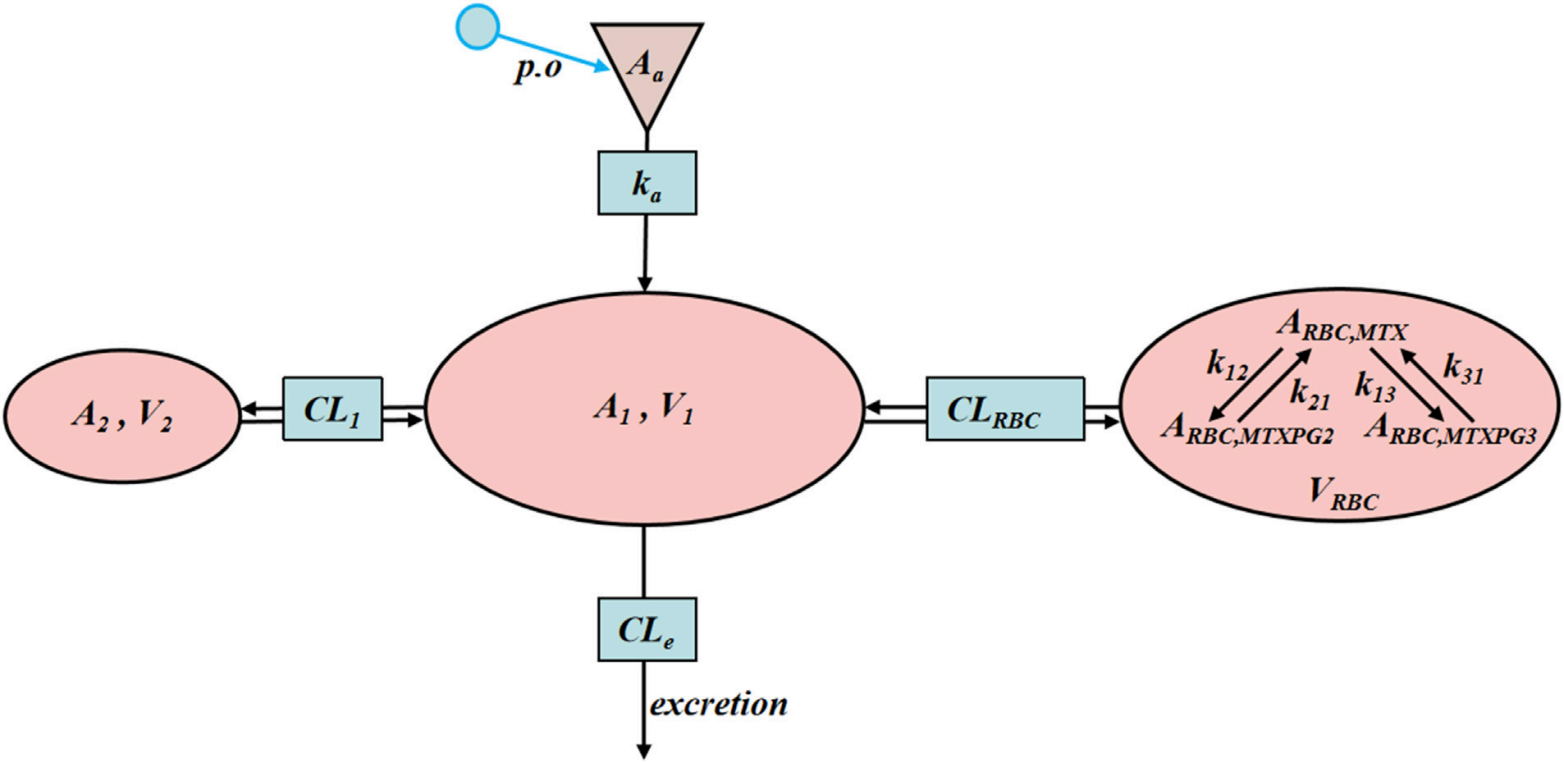
Schematic diagram of MTX *in vivo* PK model in rats.

By comparing the goodness of fit of the model, methotrexate selected a two-compartment model (2CM) that incorporates an oral absorption compartment. Additionally, a three-compartment model that includes RBC volume (V_3_) was employed to describe the conversion of MTX to MTXPG_2_ and MTXPG_3_ within RBCs, as illustrated in Figure 1. MTX present in the plasma can penetrate RBCs, forming MTXPGs, specifically MTXPG_2_ and MTXPG_3_. These compounds are then metabolized back into MTX, which is ultimately released back into the plasma.

The model equations and initial conditions are such as Equations 3- 9.

$$dA_{a}/dt=-k_{a}\times A_{a}\;A_{a\left(0\right)}=\text{Dose}$$ (3)

$$C_{1}=A_{1}/V_{1},C_{2}=A_{2}/V_{2},C_{RBC,MTX}=A_{RBC,MTX}/V_{RBC}$$ (4)

$$dA_{1}/dt=k_{a}\times A_{a}-{CL}_{e}\times C_{1}-{CL}_{2}\times \left(C_{1}-C_{2}\right)-{CL}_{RBC}\times \left(C_{1}-C_{RBC,MTX}\right)$$

$$A_{1\left(0\right)}=0$$ (5)

$$dA_{2}/dt={CL}_{2}\times \left(C_{1}-C_{2}\right)\;A_{2\left(0\right)}=0$$ (6)

$$dA_{RBC,MTX}/dt={CL}_{RBC}\times \left(C_{1}-C_{RBC,MTX}\right)-\left(A_{RBC,MTX}\times k_{12}-A_{RBC,MTXPG2}\times k_{21}\right)-\left(A_{RBC,MTX}\times k_{13}-A_{RBC,MTXPG3}\times k_{31}\right)\;A_{RBC,MTX\left(0\right)}=0$$ (7)

$$dA_{RBC,MTXPG2}/dt=\left(A_{RBC,MTX}\times k_{12}-A_{RBC,MTXPG2}\times k_{21}\right)\;A_{RBC,MTXPG2\left(0\right)}=0$$ (8)

$$dA_{RBC,MTXPG3}/dt=\left(A_{RBC,MTX}\times k_{13}-A_{RBC,MTXPG3}\times k_{31}\right)\;A_{RBC,MTXPG3\left(0\right)}=0$$ (9)

A_a_ represents the amount of MTX in the absorption compartment, which is the administered dose. *k*_a_ is the absorption rate constant. C_1_, C_2_, C_RBC,MTX_ are the drug concentrations of MTX in the central compartment (V_1_), peripheral compartment (V_2_), and RBC compartment (V_RBC_), respectively. A_1_, A_2_, and A_RBC,MTX_ are the amounts of the drug. C_RBC, MTXPG2_ and C_RBC, MTXPG3_ are the concentrations of MTXPG_2_ and MTXPG_3_ in RBC, respectively. CL_e_ is the systemic clearance rate. CL_1_ is the distribution clearance rate between V_1_ and V_2_. CL_RBC_ is the distribution clearance rate between V_1_ and V_RBC_. *k*_12_ is the rate constant for the conversion of MTX to MTXPG_2_. *k*_21_ is the rate constant for the conversion of MTXPG_2_ to MTX. *k*_13_ is the rate constant for the conversion of MTX to MTXPG_3_. *k*_31_ is the rate constant for the conversion of MTXPG_3_ to MTX.

## 3 Results

### 3.1 Concentration-time curve and PK parameters of MTX and MTXPG_2-3_

**FIGURE 2:**
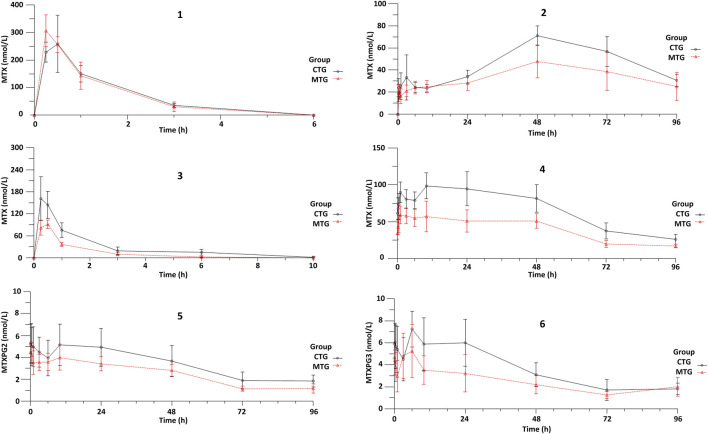
The concentration-time curve of MTX, MTXPG_2_, and MTXPG_3_ in Plasma and RBC of the CTG and MTG (1: plasma MTX of single-dose group, 2: RBC MTX of single-dose group, 3: plasma MTX of multiple-dose group, 4: RBC MTX of multiple-dose group, 5: RBC MTXPG_2_ of multiple-dose group, 6: RBC MTXPG_3_ of multiple-dose group).

The detection method developed in this study effectively identifies MTX and MTXPG_2-5_ in RBC; however, only MTX and MTXPG_2-3_ were detectable in rat erythrocytes, while MTXPG_4-5_ were below the detection limit and could not be detected. Figure 2 illustrates the concentration-time curves of MTX in both plasma and RBC for the single-dose groups (MTG and CTG), whereas the concentration-time curves of MTX, MTXPG_2_, and MTXPG_3_ in plasma and RBC from the multiple-dose groups are also displayed in Figure 2. The *C*_max_ of MTX in the single-dose group was higher (MTG: 306.3 nM, CTG: 258.9 nM) compared to that in the multiple-dose group (MTG: 91.5 nM, CTG: 161.1 nM). The metabolism of plasma MTX was rapid in both groups, with concentrations becoming undetectable after 6 and 10 h, respectively (the lower limit of quantification was 1 nM). In the RBC of the single-dose group, only MTX was detectable, with a *C*_max_ reached after 48 h (MTG: 47.8 nM, CTG: 71.1 nM). In contrast, both MTX, MTXPG_2_, and MTXPG_3_ concentrations were detectable in the RBC of the multiple-dose group. Drug concentrations in the blood remained relatively stable in the first 24 h, reaching *C*_max_ levels (MTG: MTX: 59.1 nM, MTXPG_2_: 4.5 nM, MTXPG_3_: 5.3 nM; CTG: MTX: 98.6 nM, MTXPG_2_: 5.3 nM, MTXPG_3_: 7.3 nM). Up to the conclusion of the study at 96 h, the concentrations of MTX, MTXPG_2_, and MTXPG_3_ in the RBC of both groups remained detectable, indicating a relatively slow metabolic process.

The comparisons of the AUC and PK parameters for each group are presented in Table 1. As illustrated in Figure 2, within the single-dose group, the *C*_max_ of MTX in RBC from the CTG exhibited a significant increase compared to the MTG (P = 0.023), whereas the AUC_0-last_ of MTX in RBC displayed a noticeable increasing trend (P = 0.056). In the multiple-dose group, a significant increase was observed in the *C*_max_, AUC_0-last_, and AUC_0- ∞_ of MTX in plasma for the CTG compared to the MTG (P = 0.023, P = 0.028, P = 0.024). Additionally, the *C*_max_, AUC_0-last_, and AUC_0- ∞_ of MTX in RBC from the CTG also demonstrated significant increases (P = 0.010, P = 0.003, P = 0.005). Furthermore, the *C*_max_, AUC_0-last_, and AUC_0- ∞_ of MTXPG_2_ and MTXPG_3_ in RBC from the CTG all exhibited increasing trends.

**TABLE 1:** The PK parameters of MTX, MTXPG_2_, and MTXPG_3_ in Plasma and RBC of the CTG and MTG (1: plasma MTX of single-dose group, 2: RBC MTX of single-dose group, 3: plasma MTX of multiple-dose group, 4: RBC MTX of multiple-dose group, 5: RBC MTXPG_2_ of multiple-dose group, 6: RBC MTXPG_3_ of multiple-dose group).

Group		*t*_*1/2*_ (h)	*T*_max_ (h)	*C*_max_ (nmol/L)	AUC_0-last_ (nmol/L⋅h)	AUC_0- ∞ _ (nmol/L⋅h)
1	CTG	0.97 ± 0.27	0.35 ± 0.12	265.76 ± 100.06	377.47 ± 66.14	429.87 ± 48.14
	MTG	0.85 ± 0.24	0.30 ± 0.10	386.54 ± 159.42	421.00 ± 149.87	450.41 ± 152.24
2	CTG	40.01 ± 5.87	38.45 ± 19.10	75.90 ± 6.73	4,509.59 ± 668.69	6,306.70 ± 1,074.07
	MTG	36.44 ± 16.89	57.60 ± 11.76	51.90 ± 15.75	3,303.06 ± 850.68	7,457.10 ± 4,705.03
3	CTG	1.75 ± 0.77	0.33 ± 0.12	165.53 ± 58.57	283.13 ± 134.92	304.89 ± 142.59
	MTG	1.28 ± 0.33	0.42 ± 0.12	93.20 ± 12.95	127.30 ± 18.55	133.17 ± 19.58
4	CTG	33.67 ± 5.35	14.67 ± 6.60	103.32 ± 17.79	6,495.94 ± 1,348.60	7,770.95 ± 1,663.03
	MTG	37.17 ± 6.16	6.92 ± 4.36	71.57 ± 13.62	3,822.76 ± 796.46	4,730.40 ± 888.43
5	CTG	—^a^	—^a^	5.63 ± 1.72	330.98 ± 115.12	498.66 ± 183.45
	MTG	—^a^	—^a^	4.67 ± 0.85	238.62 ± 40.37	330.27 ± 72.43
6	CTG	—^a^	—^a^	7.28 ± 1.69	352.09 ± 121.73	473.32 ± 137.66
	MTG	—^a^	—^a^	6.72 ± 1.79	226.94 ± 77.49	422.40 ± 80.80

^a^ The parameters of RBC MTXPG_2_ and RBC MTXPG_3_ cannot be obtained.

### 3.2 Concentration-time curve and PK parameters of HCQ and DHCQ

No significant differences were noted in the *C*_max_, AUC_0-last_, and AUC_0- ∞_ of HCQ and DHCQ in plasma and RBC between the HTG and CTG. However, substantial inter-individual variability was noted across all measured parameters, which compromised the goodness-of-fit in subsequent pharmacokinetic modeling analyses. This pronounced variability in HCQ and DHCQ concentrations within treatment groups may primarily stem from the inherent variability in HCQ absorption following intragastric administration. For further results, please refer to Annex 1.

### 3.3 PK model of MTX

Modeling was conducted utilizing the concentration data of MTX, MTXPG_2_, and MTXPG_3_ from blood samples of SD rats within each group. A two-compartment model with an absorption compartment was found to provide a better fit for the concentration data of plasma MTX. The physiological RBC volume for healthy SD rats was used as the volume of the third compartment, established at 0.030 L/kg. The PK model parameters for the CTG and MTG in the single-dose group are presented in Table 2. When compared to the MTG, the central compartment V_1_ for the CTG was significantly increased (P = 0.047). The PK model parameters for the CTG and MTG in the multiple-dose group are provided in Table 3. In comparison to the MTG, the V_1_ and CL_e_ for the CTG were significantly decreased (P = 0.010, P = 0.001), whereas the V_2_ was significantly increased (P = 0.016), and the *k*_*a*_ showed a tendency to increase (P = 0.074).

**TABLE 2:** PK parameters of MTX in the CTG and MTG in the single-dose group.

Parameter		MTX + HCQ		MTX		P
		Estimate	CV%	Estimate	CV%	
*k*_*a*_	1/h	6.69 ± 1.57	11.8 ± 10.8	5.22 ± 0.98	15.7 ± 7.2	0.153
V_1_	L/kg	22.70 ± 3.18	8.2 ± 11.9	14.24 ± 6.49	13.2 ± 9.2	0.047
CL_e_	L/(kg*h)	19.41 ± 1.74	18.6 ± 13.4	19.37 ± 5.91	9.9 ± 10.9	0.988
V_2_	L/kg	30.52 ± 14.01	28.8 ± 10.0	28.04 ± 27.46	13.0 ± 6.2	0.881
CL_1_	L/(kg*h)	18.86 ± 17.00	32.2 ± 26.8	11.64 ± 6.75	27.8 ± 27.4	0.579
V_RBC_	L/kg	0.030	Fixed^a^	0.030	Fixed^a^	—
CL_RBC_	L/(kg*h)	0.00506 ± 0.00565	26.0 ± 20.1	0.00219 ± 0.00020	33.4 ± 18.8	0.341

^a^ Parameter fixed to value scaled from rats data in literature.

**TABLE 3:** PK parameters of MTX in the CTG and MTG in the multiple-dose group.

Parameter		MTX + HCQ		MTX		P
		Estimate	CV%	Estimate	CV%	
*k*_*a*_	1/h	6.45 ± 3.36	22.3 ± 7.8	3.38 ± 0.67	24.7 ± 8.6	0.074
V_1_	L/kg	19.23 ± 1.87	12.8 ± 8.9	36.58 ± 9.21	16.2 ± 5.9	0.010
CL_e_	L/(kg*h)	22.93 ± 9.38	10.3 ± 8.1	72.03 ± 13.66	21.2 ± 7.3	0.001
V_2_	L/kg	110.99 ± 47.90	26.9 ± 16.1	45.6 ± 9.79	18.5 ± 9.3	0.016
CL_1_	L/(kg*h)	28.44 ± 12.09	22.1 ± 12.6	21.53 ± 12.73	20.3 ± 13.9	0.465
V_RBC_	L/kg	0.030	Fixed^a^	0.030	Fixed^a^	
CL_RBC_	L/(kg*h)	0.0355 ± 0.0171	25.7 ± 13.8	0.0290 ± 0.0147	28.9 ± 8.9	0.637
*k*_*12*_	1/h	0.00417 ± 0.00146	—^b^	0.00753 ± 0.00185	—^b^	0.021
*k*_*21*_	1/h	0.000773 ± 0.000280	—^b^	0.000708 ± 0.000315	—^b^	0.766
*k*_*13*_	1/h	0.00612 ± 0.00238	—^b^	0.00738 ± 0.00315	—^b^	0.550
*k*_*31*_	1/h	0.00168 ± 0.0069	—^b^	0.00173 ± 0.00157	—^b^	0.951

^a^ Parameter fixed to value from rats data in literature.

^b^ Secondary parameter, coefficient of variation could not be calculated.

## 4 Discussion

The *C*_max_ and AUC of CTG in plasma and RBC were significantly greater than those of MTG, indicating that HCQ influences the PK behavior of MTX in rats. However, there were no significant differences in the *C*_max_ and AUC of HCQ and its metabolite, DHCQ, in the plasma and RBC of rats under any administration conditions. This suggests that MTX does not impact the metabolism and absorption of HCQ, a finding consistent with the clinical research conducted by Carmichael et al. (2002).

MTX is primarily transported into cells through active uptake mechanisms involving transporters such as the reduced folate carrier 1 (RFC1/SLC19A1) and the organic anion transporting polypeptides (OATPs) (Tang et al., 2024). Once inside the cell, additional glutamate residues are added by folate polyglutamate synthetase (FPGS) to form MTXPGs that accumulate intracellularly. This process can be reversed by γ-glutamyl hydrolase (GGH), which converts MTXPGs back to their monoglutamate form. Subsequently, these monoglutamate MTXPGs are extruded from the cell via efflux transporters, including multidrug resistance protein 1 (MDR1/ABCB1), multidrug resistance-associated protein 1 (MRP1), and breast cancer resistance protein (BCRP) (Stamp et al., 2013). Research indicates that HCQ does not exhibit inhibitory effects on MRP1 and OATPs such as OATP1B1, OATP1B3, and OATP2B1, nor does it stimulate the regulation of the ABCB1 gene. However, HCQ can inhibit ABCB1 and reduce the efflux of MTX when the concentration exceeds 10 μM (Weiss et al., 2020). This is consistent with the notable increase in the *C*_max_ of MTX in RBC of the CTG during single-dose administration, as well as the upward trend in AUC_0-last_. The significant increases in the *C*_max_, AUC_0-last_, and AUC_0- ∞_ of MTX in RBC of the CTG during multiple-dose administration further corroborate that the exposure level of MTX within RBC has risen markedly. Furthermore, the levels of MTXPG_2_ and MTXPG_3_ have also increased to a certain extent. Although this study suggests that HCQ may increase systemic exposure to MTX by inhibiting ABCB1, the sensitivity of human ABCB1 to HCQ may be different from that of rats, and its clinical translational value should be carefully evaluated. Future studies need to validate ABCB1-mediated MTX-HCQ interactions in human primary hepatocytes or intestinal models, and further confirm the association with clinical monitoring data such as blood concentrations of HCQ and MTX in RA patients.

Most of MTX is excreted in its original form, with approximately 75% eliminated through renal tubular secretion. During the process of renal tubular secretion, OATPs and RFC-1 transport MTX from the bloodstream to the basolateral membrane. Subsequently, ABCB1, MRP1, and BCRP transport MTX across the apical membrane into the urine and out of the body (Iwaki et al., 2017). Therefore, the concurrent use of ABCB1 inhibitors may elevate the plasma levels of MTX in both plasma and tissues, potentially enhancing its efficacy as well as toxicity (Liu, 2019). In the context of multiple-dose administration, *C*_max_, AUC_0-last_, and AUC_0- ∞_ of plasma MTX in the CTG all exhibited significant increases, whereas CL_e_ decreased markedly. These alterations indicate that combined use with HCQ significantly raised the plasma exposure levels of MTX. This effect may be attributed to HCQ’s inhibition of ABCB1, which reduces MTX clearance and enables sustained higher plasma concentrations of the drug. Additionally, this interaction allows for greater uptake and transport of MTX into RBC.

Modeling the plasma concentration data of MTX revealed that a two-compartment model with an absorption compartment better captures its variation pattern. RBCs can act as a drug reservoir, influencing the corresponding drug concentration at the target site. By including them as an independent third compartment in the model, the simulation of drug distribution in the body becomes more accurate and aligns more closely with physiological processes. In the case of multiple-dose administration within the CTG, significant decreases in V_1_ and CL_e_ were noted, along with a substantial increase in V_2_ and a tendency for *k*_a_ to rise. The decrease in V_1_ indicates an increase in the distribution of the drug within the central compartment, whereas the concurrent increase in V_2_ suggests a decrease in drug distribution in the peripheral compartment. This indicates a significant alteration in drug distribution within the body, characterized by a greater concentration in the central compartment and a reduction in tissue distribution. The notable decrease in CL_e_ implies a reduction in the drug clearance rate, indicating that HCQ may inhibit the body’s ability to eliminate MTX, thereby prolonging its presence in the body. The observed tendency for *k*_a_ to increase suggests that HCQ may enhance the intestinal absorption of MTX.

In conclusion, the use of combination therapy has enhanced the bioavailability of MTX and increased its exposure in SD rats. These results may explain why the combination of MTX-HCQ in clinical practice results in improved efficacy, a faster onset of action, and a longer duration of continuous effect compared to MTX monotherapy. The alterations in PK parameters of MTX observed in this study are significant for its clinical application. Both single-dose and multiple-dose administrations of MTX in the CTG showed significant increases in *C*_max_ and AUC in the blood. Given that HCQ may inhibit kidney transporters involved in MTX elimination, patients with renal insufficiency may be at greater risk of toxicity due to reduced drug clearance (Wang et al., 2018). Our data suggest that co-administration of HCQ-MTX may exacerbate systemic accumulation in these populations, requiring dose adjustment and rigorous TDM. It is recommended to be alert for adverse reactions during combined therapy with methotrexate and HCQ, and to monitor blood concentrations if necessary, especially in patients with renal insufficiency. Future clinical trials should validate these PK interactions and establish evidence-based dose-adjustment protocols for at-risk populations.

This study elucidates MTX-HCQ pharmacokinetic interactions but has limitations: 1) Healthy rat models differ pathophysiologically from rheumatoid arthritis patients; 2) Tissue distribution (e.g., synovium) and transporter activity were not directly measured; 3) Short-term dosing may underestimate cumulative toxicity; 4) Rodent-human extrapolation requires PBPK/clinical validation. Translational studies integrating human cell models and therapeutic monitoring are needed.

## 5 Conclusion

In summary, this research provides valuable insights into the clinical benefits associated with combination therapies. The findings may clarify the reasons behind the enhanced efficacy, faster onset of action, and prolonged duration of effect observed with the combination of MTX and HCQ, as compared to monotherapy with MTX alone. Given the significant increases in *C*_max_ and AUC, it is crucial to remain particularly vigilant regarding the adverse reactions of MTX during treatment with HCQ, especially in patients with renal insufficiency. Future research should focus on elucidating the mechanisms underlying the drug-drug interactions identified in this study. Furthermore, exploring the potential impact of combination therapies on patient outcomes, including efficacy, toxicity, and overall survival, would be beneficial.

## Data Availability

The original contributions presented in the study are included in the article/Supplementary Material, further inquiries can be directed to the corresponding author.
