# Supplementary material for: Hydroxychloroquine increases the exposure of methotrexate in plasma and red blood cells: a pharmacokinetic interaction study in rats in vivo
Source: Front Pharmacol. 2025 Apr 24;16:1561001. doi: 10.3389/fphar.2025.1561001 (PMC12059348; doi:10.3389/fphar.2025.1561001)
Supplement: Supplementary file 1 [file DataSheet1.docx]

Supplementary Materials for

**Hydroxychloroquine increases the exposure of methotrexate in plasma and red blood cells: A Study in Rats In Vivo**

Guijie Zhang^a^, Rui Wang^a^, Geping Chen^a^, Simin Liu^b^, Hongyu Jie^c^, Wenying Chen^a^, Qiang Li^a^*

*^a^ Department of Pharmacy, The Third Affiliated Hospital of Southern Medical University, Guangzhou, Guangdong 510630, China*

*^b^ School of Pharmaceutical Sciences, Southern Medical University, Guangzhou, Guangdong 510511, China*

*^c^ Department of Rheumatology and Immunology, The Third Affiliated Hospital of Southern Medical University, Guangzhou, Guangdong 510630, China*

*1. Bioanalytical methodology*

*Table 1-1.* Gradient elution program for online MTX, MTXPG_2_, and MTXPG_3_ analysis

| Left pump/Analysis pump | | | | Right pump/Extraction pump | | | |
| --- | --- | --- | --- | --- | --- | --- | --- |
| Time/min | Flow rate mL/min | A1 | B1 | Time/min | Flow rate mL/min | A2 | B2 |
| 0.00 | 1.0 | 98% | 2% | 0.00 | 1.5 | 100% | 0% |
| 1.00 | 1.0 | 98% | 2% | 2.00 | 1.5 | 100% | 0% |
| 6.00 | 1.0 | 94% | 6% | 2.10 | 1.5 | 100% | 0% |
| 13.00 | 1.0 | 86% | 14% | 2.10 | 0.1 | 100% | 0% |
| 14.00 | 1.0 | 15% | 85% | 12.00 | 0.1 | 100% | 0% |
| 15.00 | 1.0 | 15% | 85% | 12.00 | 1.0 | 100% | 0% |
| 16.00 | 1.0 | 98% | 2% | 13.00 | 1.0 | 15% | 85% |
|  |  |  |  | 15.00 | 1.0 | 15% | 85% |
|  |  |  |  | 16.00 | 1.0 | 100% | 0% |
|  |  |  |  | 17.00 | 1.0 | 100% | 0% |
|  |  |  |  | 17.00 | 1.5 | 100% | 0% |
| 18.00 | Stop running | | | 18.00 | Stop running | | |

*Table 1-2.* Gradient elution program for online HCQ and DHCQ analysis

| Left pump/Analysis pump | | | | Right pump/Extraction pump | | | |
| --- | --- | --- | --- | --- | --- | --- | --- |
| Time/min | Flow rate mL/min | A1 | B1 | Time/min | Flow rate mL/min | A2 | B2 |
| 0 | 1.0 | 93% | 7% | 0 | 1.0 | 98% | 2% |
| 3.50 | 1.0 | 93% | 7% | 3.50 | 1.0 | 98% | 2% |
| 9.00 | 1.0 | 85% | 15% | 5.00 | 1.0 | 20% | 80% |
| 11.00 | 1.0 | 20% | 80% | 6.00 | 1.0 | 98% | 2% |
| 12.00 | 1.0 | 93% | 7% | 6.00 | 0.1 | 98% | 2% |
|  |  |  |  | 12.00 | 0.1 | 98% | 2% |
|  |  |  |  | 12.00 | 1.0 | 98% | 2% |
| 14.00 | Stop running | | | 14.00 | Stop running | | |

1. *Concentration-time curve and PK parameters of HCQ and DHCQ*

| *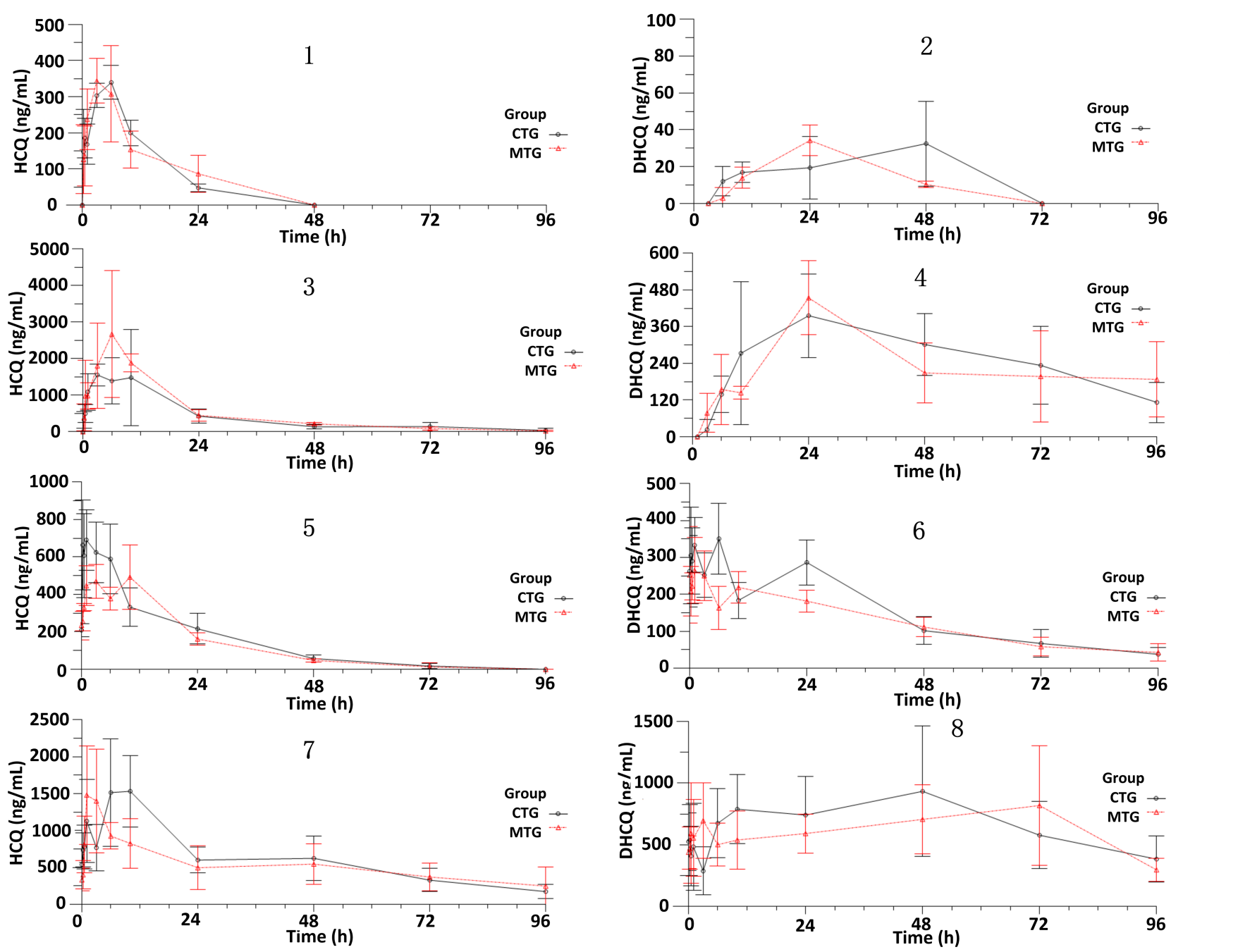* |
| --- |

*Figure 1.* The concentration-time curve of HCQ and DHCQ in Plasma and RBC of the CTG and MTG (1: plasma HCQ of single-dose group, 2: plasma DHCQ of single-dose group, 3: RBC HCQ of single-dose group, 4: RBC DHCQ of single-dose group, 5: plasma HCQ of multiple-dose group, 6: plasma DHCQ of multiple-dose group, 7: RBC HCQ of multiple-dose group, 8: RBC DHCQ of multiple-dose group).

*Table 2-1.* The PK parameters of HCQ and DHCQ in Plasma and RBC of the CTG and MTG (1: plasma HCQ of single-dose group, 2: plasma DHCQ of single-dose group, 3: RBC HCQ of single-dose group, 4: RBC DHCQ of single-dose group, 5: plasma HCQ of multiple-dose group, 6: plasma DHCQ of multiple-dose group, 7: RBC HCQ of multiple-dose group, 8: RBC DHCQ of multiple-dose group).

| Group | | *t1/2*（h） | *Tmax*（h） | *Cmax*（ng/mL） | AUC0-last（ng/mL*h） | AUC0-∞（ng/mL*h） |
| --- | --- | --- | --- | --- | --- | --- |
| 1 | CTG | 6.52±0.89 | 5.20±1.20 | 348.60±44.52 | 4394.00±562.03 | 4851.42±607.66 |
|  | HTG | 12.22±4.87 | 4.20±1.47 | 368.84±92.11 | 4303.84±1011.52 | 6144.09±2730.01 |
| 2 | CTG | 13.81±3.97 | 43.20±9.60 | 35.04±20.80 | 1150.62±978.70 | 1431.89±967.62 |
|  | HTG | 15.33±4.59 | 24.00±0.00 | 34.22±8.20 | 909.09±183.42 | 1142.15±149.98 |
| 3 | CTG | 26.75±14.18 | 5.60±2.58 | 2273.12±955.42 | 37579.20±15388.28 | 44748.45±16783.05 |
|  | HTG | 19.15±3.04 | 7.00±2.68 | 3453.52±1361.60 | 48252.09±11518.74 | 49696.67±11340.82 |
| 4 | CTG | 36.76±14.47 | 30.80±14.95 | 449.94±168.60 | 24707.65±7638.65 | 31727.46±11437.26 |
|  | HTG | 21.72±14.21 | 33.60±19.20 | 467.78±117.74 | 22645.53±2990.47 | 27959.11±2265.08 |
| 5 | CTG | 15.73±1.73 | 1.38±2.09 | 867.60±100.21 | 13374.34±3133.35 | 14181.09±3151.58 |
|  | HTG | 13.43±3.88 | 4.33±4.07 | 592.05±110.87 | 11721.22±1885.82 | 12572.99±2060.21 |
| 6 | CTG | 25.02±4.43 | 2.45±2.52 | 456.41±78.87 | 14098.40±2796.74 | 15570.35±3574.36 |
|  | HTG | 34.72±14.57 | 1.17±1.34 | 322.55±93.38 | 11722.63±1765.11 | 14363.27±4359.29 |
| 7 | CTG | 35.26±16.84 | 10.08±7.10 | 2003.93±477.12 | 63284.49±20586.02 | 73718.38±23635.83 |
|  | HTG | 49.36±30.11 | 11.00±16.82 | 1829.77±649.18 | 50941.66±11574.11 | 67010.61±15406.92 |
| 8 | CTG | 43.35±19.24 | 21.21±20.50 | 1307.23±189.13 | 66082.85±18930.12 | 91518.60±31376.07 |
|  | HTG | 47.80±20.09 | 41.75±27.74 | 1150.08±188.50 | 60804.84±12722.65 | 81560.94±25650.06 |
